# Repression of Osteoblast Maturation by ERRα Accounts for Bone Loss Induced by Estrogen Deficiency

**DOI:** 10.1371/journal.pone.0054837

**Published:** 2013-01-24

**Authors:** Marlène Gallet, Soraya Saïdi, Eric Haÿ, Johann Photsavang, Caroline Marty, Juliette Sailland, Julie Carnesecchi, Violaine Tribollet, Bruno Barenton, Christelle Forcet, Marie-Christine Birling, Tania Sorg, Olivier Chassande, Martine Cohen-Solal, Jean-Marc Vanacker

**Affiliations:** 1 Institut de Génomique Fonctionnelle de Lyon, Université de Lyon, Centre national de la recherche scientifique UMR5242, Ecole Normale Supérieure de Lyon, Lyon, France; 2 Institut National de la Santé et de la Recherche Médicale U606, Hôpital Lariboisière, Paris, France; 3 Institut National de la Santé et de la Recherche Médicale U1026, Bordeaux, France; 4 Institut Clinique de la Souris, Illkirch-Graffenstaden, France; The Chinese University of Hong Kong, Hong Kong

## Abstract

ERRα is an orphan member of the nuclear receptor family, the complete inactivation of which confers resistance to bone loss induced by ageing and estrogen withdrawal to female mice in correlation with increased bone formation *in vivo*. Furthermore ERRα negatively regulates the commitment of mesenchymal cells to the osteoblast lineage *ex* vivo as well as later steps of osteoblast maturation. We searched to determine whether the activities of ERRα on osteoblast maturation are responsible for one or both types of *in vivo* induced bone loss. To this end we have generated conditional knock out mice in which the receptor is normally present during early osteoblast differentiation but inactivated upon osteoblast maturation. Bone ageing in these animals was similar to that observed for control animals. In contrast conditional ERRαKO mice were completely resistant to bone loss induced by ovariectomy. We conclude that the late (maturation), but not early (commitment), negative effects of ERRα on the osteoblast lineage contribute to the reduced bone mineral density observed upon estrogen deficiency.

## Introduction

Bone remodeling is a dynamic process in which resorption exerted by osteoclasts is compensated for by formation exerted by osteoblasts. Although normally tightly regulated, this equilibrium can be disrupted under various circumstances [1], [2]. For instance, during ageing mesenchymal cells (MCs, the osteoblast progenitors) become more prone to differentiate into adipocytes than into osteoblast, *in fine* leading to a relative excess osteoclast activity. In contrast estrogen deficiency in post-menopause women results in derepressed osteoclast differentiation and activity that is not compensated for by a similarly increased osteoblast activity. Ageing and menopause thus both lead to increased bone fragility (*i.e.* osteoporosis) and enhanced fracture risk. Given the global ageing of the world population, osteoporosis and its consequences now represent a major public health problem, in particular in females. Treatments against post-menopause osteoporosis have up to now generally aimed at reducing osteoclast activities, in particular via hormone replacement therapies [3]–[5]. Due to controversial side effects of the latter treatments, the possibility of enhancing osteoblast differentiation and/or activities (bone anabolism) could appear as a promising approach [6], [7].

MCs differentiation into osteoblast has increasingly been characterized including at the level of the sequential expression of functional markers. Two transcription factors, Runx2 and Osx, are instrumental in the first steps of this cascade and precede and/or regulate the expression of later markers, which reflect osteoblast maturation. These include collagen 1a (Col1a), alkaline phosphatase (ALP), osteocalcin and osteopontin [8].

ERRα belongs to the nuclear receptor family, the members of which act as transcription factors [9]. Although no natural ligand has been identified to date for ERRα (which is thus referred to as “orphan receptor”), synthetic compounds have been isolated that modulate its activities and/or protein stability [10]–[13]. High expression of ERRα in various human cancer types is correlated to poor prognosis (reviewed in [14]). Furthermore the receptor has been shown to promote tumorigenicity and angiogenesis in human carcinoma cells xenografted onto Nude mice ([15]–[17], reviewed in [18]), suggesting that inhibition of ERRα could be beneficial in cancer treatment [19]. Several reports have demonstrated that ERRα is a key positive regulator of various metabolic functions, controlling lipid uptake, fatty acid oxidation, the tricarboxylic acid cycle, oxidative phosphorylation and mitochondrial biogenesis (reviewed in [20], [21]). In addition, ERRα impacts on MCs differentiation, promoting commitment towards the adipocyte pathway [22] while inhibiting the osteoblast one [23], [24]. Indeed, when set in differentiation *ex vivo*, MCs originating from ERRα knock-out (ERRαKO) mice display a higher and earlier expression of Runx2, Col1a, ALP and osteocalcin, associated with increased mineralization capacities. Mechanistically ERRα has been suggested to modulate Wnt signaling [25] whereas the closely related ERRγ receptor (which also represses osteoblast differentiation) impairs Runx2 transcriptional activities [26]. In addition to these early effects on MCs commitment, ERRα may impact on later steps of osteoblast differentiation. Indeed, the enhanced *ex* vivo differentiation of ERRαKO-originating MCs can be reverted by re-expression of the receptor after the onset of differentiation, suggesting that ERRα-deficient MCs still display a certain level of plasticity [24]. Furthermore, the expression of osteopontin, an inhibitor of mineralization and late marker of osteoblast differentiation, is down regulated in ERRα-deficient osteoblasts, in contrast to other earlier markers. *In vivo* studies have shown that the bone mineral density of female ERRαKO mice is not reduced upon ageing, in contrast to the situation in wild type animals [23], [24]. Furthermore, these mice also resist to the bone loss resulting from estrogen withdrawal (obtained by ovariectomy). These phenomena are associated with an increased bone formation rate without any modification in bone resorption, indicating that osteoblasts, not osteoclasts, are the major cellular effectors mediating these resistances. In contrast, male ERRαKO mice were indistinguishable from wild type animals both in terms of bone ageing and sensitivity to androgen deficiency. Inactivating ERRα may thus be beneficial in females to protect bone against the deleterious impacts of ageing and estrogen deficiency by promoting bone anabolism (reviewed in [27]).

It is unknown whether only one or both the early and late (as defined above) effects of ERRα on osteoblast differentiation contribute to which *in vivo* resistance. To investigate this question, we have used the Cre-Lox technology to engineer mice in which ERRα is specifically inactivated during the late phase of osteoblast differentiation. We here report that female conditional ERRαKO mice resist to bone loss induced by ovariectomy but not to the one induced by natural ageing. ERRα thus contributes to estrogen deficiency-induced bone loss through its inhibitory activities on osteoblast maturation. Moreover, our results describe an animal model in which age-related and hormone-deficiency-related bone loss in females can be clearly uncoupled.

## Results

**Figure 1:**
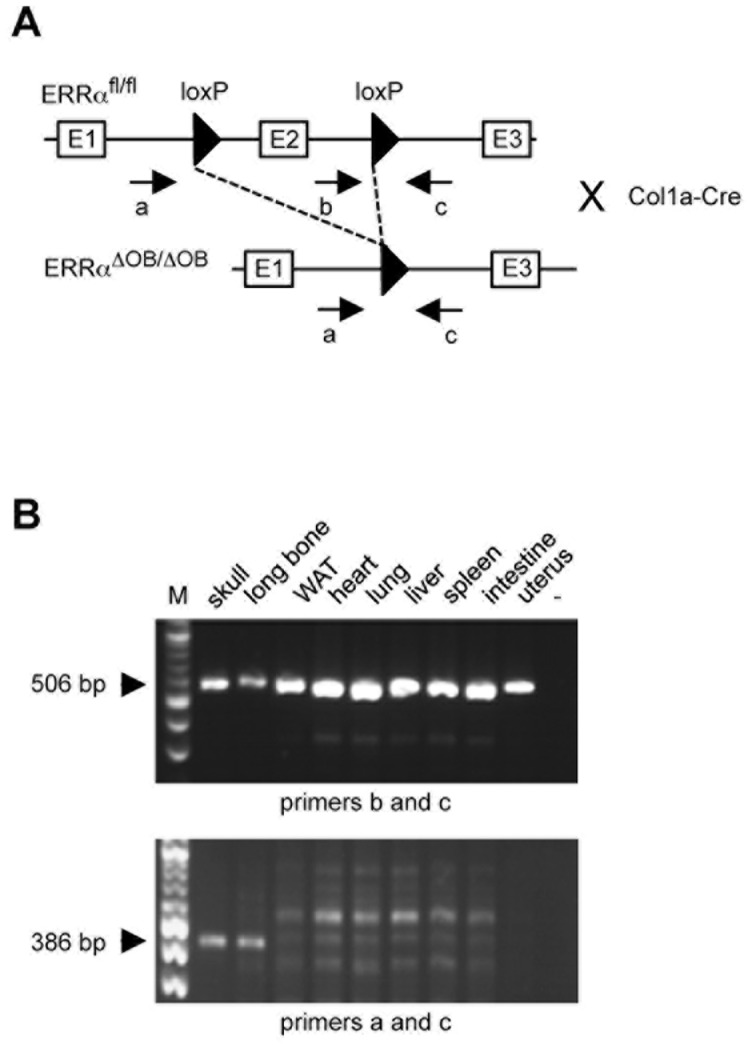
Validation of ERRα conditional knock-out approach. **A.** General strategy used. Exon 2 (E2; encoding the first zinc finger in the DNA binding domain) of the ERRα gene was flanked by loxP sites in ERRα^fl/fl^ mice (here after referred to as control [c] mice). Crossing these mice with Col1a-Cre animals resulted in ERRαΔ^OB^Δ^OB^,Col1a^Cre/+^ mice (hereafter referred to as conditional knock-out [cKO] animals), which display E2 deletion specifically in maturing osteoblasts. Location of PCR primers (see sequences in Material and Methods section) for the detection of the various alleles is depicted. Note that the gene structure is not drawn to scale. **B.** Genotyping of ERRα alleles. PCR using the indicated primers were performed using DNA extracted from the indicated organs. Upper panel, detection of the floxed allele (506 bp) in all organs tested; lower panel, detection of the recombinant allele (386 bp) in skull vault and long bone. M: size marker; −: blank PCR (no DNA).

To generate a conditional knock-out mouse model for ERRα, we used ERRα^fl/fl^ mice in which ERRα exon 2 was flanked by loxP sites. These animals were crossed with Col1a-Cre mice [28]. The latter animals express the Cre recombinase under the control of a collagen 1a promoter fragment, which is selectively active in early osteoblasts, only after the onset of differentiation. The resulting mice (ERRαΔ^OB^Δ^OB^, Col1a^Cre/+^) are thus predicted to be inactivated for ERRα only in maturing osteoblasts and not in mesenchymal stem cells or early committed osteoblast progenitors (Fig. 1A). Genotyping using specific primer sets detected the floxed allele in all tissues tested, whereas recombination only occurred in bone (long bone and skull) but not in “soft” (*i.e.* bone-free) tissues (Fig. 1B). This recombination appears only partial at the organ level likely due to the unavoidable presence of osteoclasts and non-bone contaminating cells (*e.g.* blood cells) as well as to the heterogeneity in the stages of maturation in which osteoblasts are in bone. ERRα^fl/fl^ and ERRαΔ^OB/^Δ^OB^,Col1a^Cre/+^ mice are hereafter referred to as control and conditional knock-out (cKO) mice, respectively.

**Figure 2:**
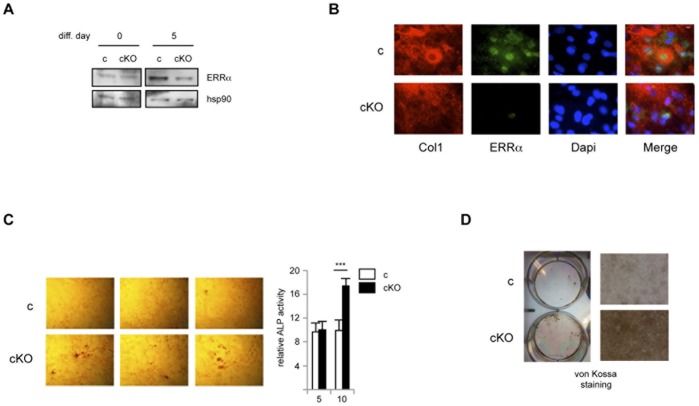
Conditional inactivation of ERRα enhances late osteoblast functions. Pre-osteoblasts from control (c) or conditional knock-out (cKO) mice were isolated from calvaria and allowed to differentiate *ex vivo*. **A.** Expression of ERRα detected in western blot after the indicated differentiation period. Hsp90 was used as a loading control. **B.** Lack of ERRα expression in Col1a-expressing cKO cells. After 6 days of differentiation, cells were fixed and stained with Dapi, anti-Col1a or anti-ERRα antibodies as indicated. **C.** Alkaline phosphatase activity was detected by whole cell cultures staining after 10 days of differentiation (left panel), or by *in vitro* enzymatic assay relative to protein content (right panel) after the indicated days of differentiation. The latter panel depicts a typical experiment (out of three) performed in triplicate. Data are expressed as average with error bars representing S.D. **D.** Mineralization activity was detected by von Kossa staining after 15 days of differentiation. Whole culture wells are presented on the left panels, field enlargements on the right ones.

Due to the above-mentioned tissue heterogeneity, a decrease of ERRα expression could not be measured in bone *in vivo*. We thus turned to an *ex vivo* model in which pre-osteoblasts from mice calvaria (cranial vault) were set in culture and allowed to differentiate into osteoblasts. At the beginning of differentiation, ERRα protein was equally (albeit weakly) detected in cells from control and cKO mice (Fig. 2A). After five days of differentiation, ERRα expression was enhanced in control cells as expected [29] whereas it was dramatically lower in cKO-originating cells. ERRα loss appeared only partial, which can be due to the heterogeneity of the cell culture that also contains fibroblasts. We thus examined the expression of ERRα by immunofluorescence in cells expressing Col1a (*i.e.* differentiating osteoblasts) (Fig. 2B). In control cultures, ERRα was expressed in the nucleus of these cells, as expected. However, in cKO-originating cells, the receptor was undetectable in Col1a-expressing cells, demonstrating a complete deletion of ERRα in maturing osteoblasts.

**Figure 3:**
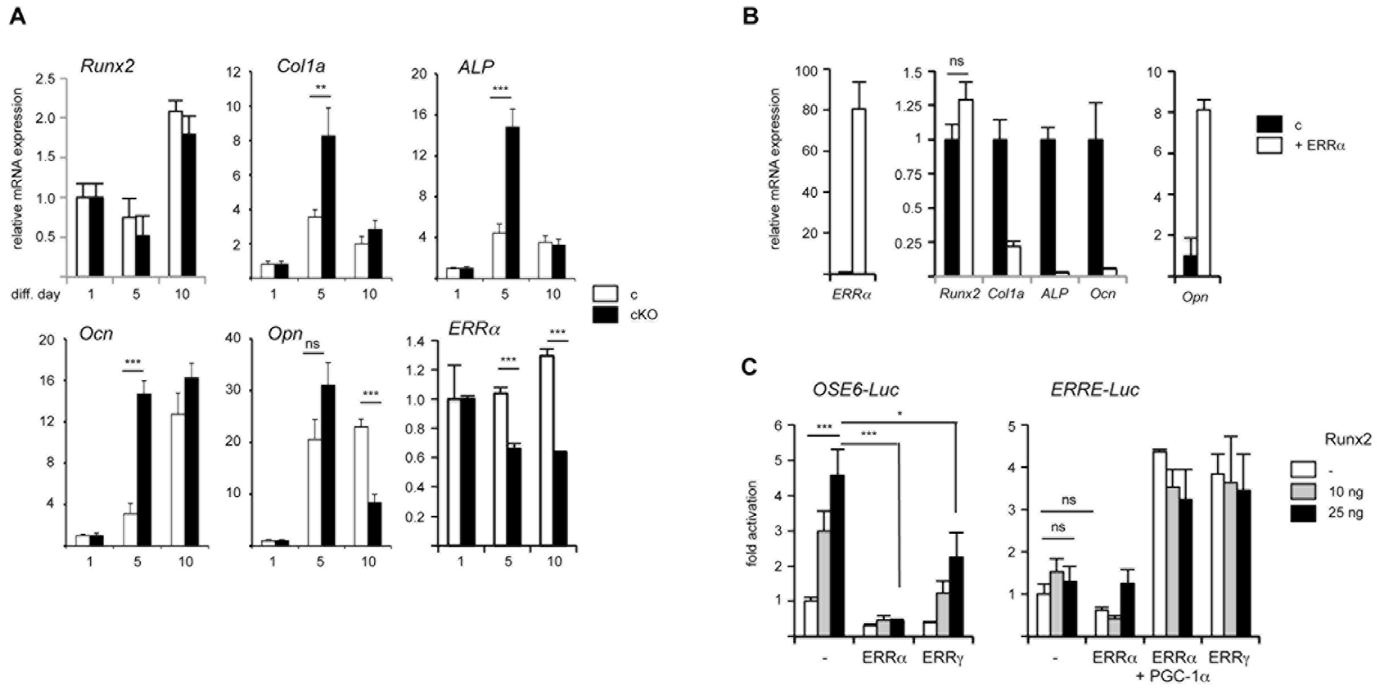
ERRα represses osteoblast differentiation markers. **A.** Evaluation of the expression of osteoblast differentiation markers by real time PCR at the indicated time after differentiation of mouse calvarial cells. ALP: alkaline phosphatase; Ocn: osteocalcin; Opn: osteopontin. A single representative experiment (out of three, performed in triplicate) is shown. Data are expressed relative to differentiation day 1 with error bars representing S.D. **B.** Reintroduction of ERRα in cKO cells normalizes the expression of differentiation parameters. Lentivirus encoding human ERRα was used to infect mouse calvarial cells induced in osteoblastic differentiation. RNAs were extracted after differentiation day 5. Data are expressed relative to control-infected cells (empty lentivirus: c) as mean +/− s.e.m. Differences for each gene are significant (*p*<0.005) except where indicated. Experiments were performed in triplicate with n = 4. **C.** ERRα represses Runx2 transcriptional activities. C3H10T1/2 cells were transfected with OSE6-Luc (Runx2-responsive) or ERRE-Luc (ERRα-responsive) plasmid together with the indicated amount of Runx2, and fixed amounts of ERRα or ERRγ, as indicated. CMV-renillaLuc was added as a transfection efficiency control and PGC-1α-encoding plasmid was added together with ERR for the right panel as indicated. Results are expressed as fold activation over transfection with activator and expressed as the average of a typical triplicate experiment (out of three) with error bars representing S.D. *: *p*<0.05; **: *p*<0.01; ***: *p*<0.005; ns: non significant.

We next analyzed the differentiation parameters of these cells. We found that, in the absence of ERRα (cKO-originating cells), alkaline phosphatase (ALP) activity was enhanced as compared to control cells, as evidenced by a greater number of labeled foci and global enzymatic activity (Fig. 2C). Mineralizing activity was also dramatically enhanced in cKO cultures as compared to control ones (Fig. 2D). In the absence of ERRα, the expression (measured by real time PCR) of middle to late differentiation markers such as Col1a, ALP and osteocalcin (Ocn) was transiently enhanced (after 5 days of differentiation) and thereafter normalized, suggesting a time-dependent effect of the receptor (Fig. 3A). In contrast, expression of osteopontin, an ERRα direct target gene [30], was reduced after 10 days of differentiation. Interestingly these variations of expression are identical to those observed when analyzing cells originating from complete (*i.e.* not conditional) ERRαKO mice [24]. Reintroduction of ERRα by lentiviral infection reversed the expression of the differentiation markers (Fig. 3B). However, the expression of Runx2, an early differentiation inducer (the expression of which precedes that of Col1a; [8]) was not modified in cKO-originating cells as compared to control ones nor upon reintroduction of ERRα. Since Runx2 activates the expression of Col1a, ALP and Ocn [8], this may altogether suggest that ERRα counteracts Runx2 activity. In this hypothesis, ERRα would act similarly to the closely related ERRγ [26]. To investigate this possibility, we performed cotransfection experiments in C3H10T1/2 (mesenchymal) cells (Fig. 3C). Runx2 activated expression driven by its cognate response element (OSE; [31]). However coexpression of ERRα or ERRγ completely blunted this effect. As a control, we verified that both ERRγ and ERRα were capable of activating transcription from their common response element (ERRE), even if ERRα requires the presence of the PGC-1α coactivator in these cells. Noteworthy, Runx2 did not impact on ERR activities under these conditions. Similar results were obtained in C2C12 cells, which are more committed than C3H10T1/2 (data not shown).

Altogether this shows that *i)* ERRα is specifically inactivated in cKO cells only after the onset of osteoblast differentiation *ex vivo*, *ii)* the absence of the receptor under these conditions promotes late osteoblast differentiation without impacting on early commitment and differentiation steps. This validates the present cKO model as a tool to study the *in vivo* effects of ERRα on osteoblast maturation (as opposed to early MSC commitment).

**Figure 4:**
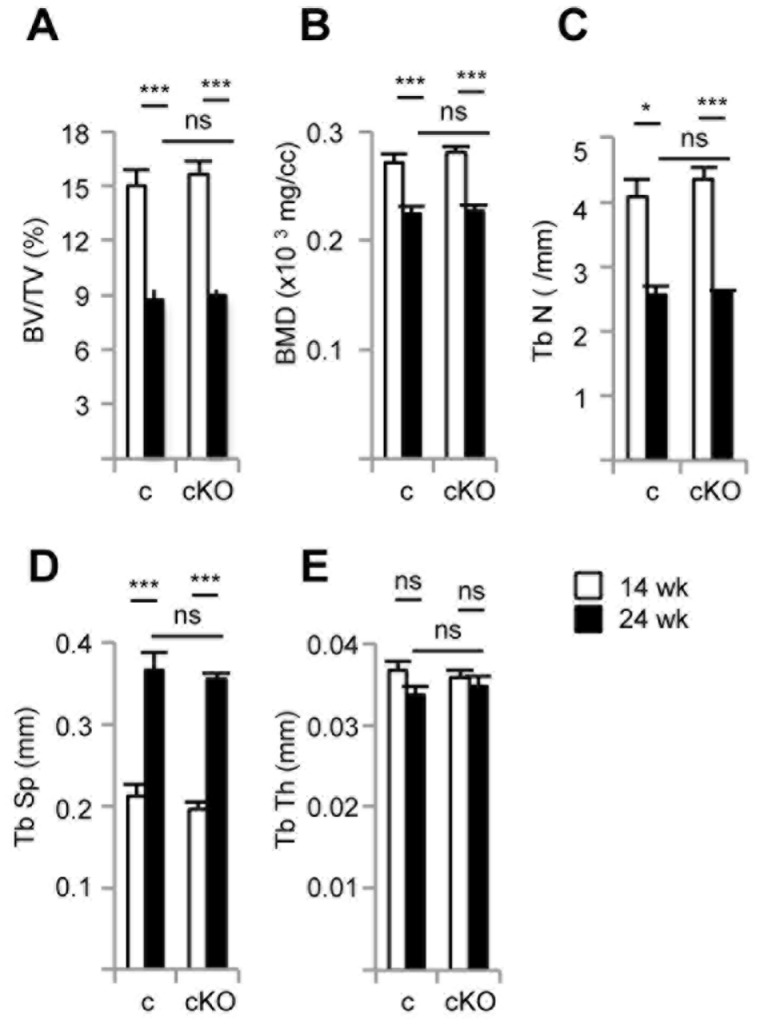
Conditional inactivation of ERRα *in vivo* does not impact bone ageing. Bone volume fraction (BV/TV; **A**), bone mineral density (BMD; **B**), trabecular number (Tb N; **C**), trabecular spacing (Tb Sp; **D**) and trabecular thickness (Tb Th; **E**) were determined by microCT-scan at 14 (white bars) and 24 wk (black bars) in the femur of female control (c) and conditional ERRα knock-out (cKO) mice (n = 6 to 11 per group). Error bars represent s.e.m. *: *p*<0.05; ***: *p*<0.005; ns: non significant.

The absence of ERRα in complete KO mice protects female (but not male) animals against age-induced as well as against ovariectomy-induced bone loss [24]. We thus first investigated whether cKO mice were also protected from age-related bone loss. To this end bone structural parameters were determined by X-ray microtomography (microCt) comparing 14 wk and 24 wk old females (Fig. 4). We observed an equal reduction of bone volume (BV/TV, Fig. 4A), bone mineral density (BMD, Fig. 4B) and trabecular number (Tb N, Fig. 4C) in both genotypes upon ageing. Trabecular spacing (Tb Sp, Fig. 4D) was also equally enhanced, reflecting the decreased number of trabeculae, in spite of constant trabecular thickness (Tb Th, Fig. 4E), not expected to decrease upon ageing. Noteworthy all these parameters displayed identical values between control and cKO mice at a given age, indicating that ERRα inactivation after the onset of osteoblast differentiation does not protect against age-induced bone loss.

**Figure 5:**
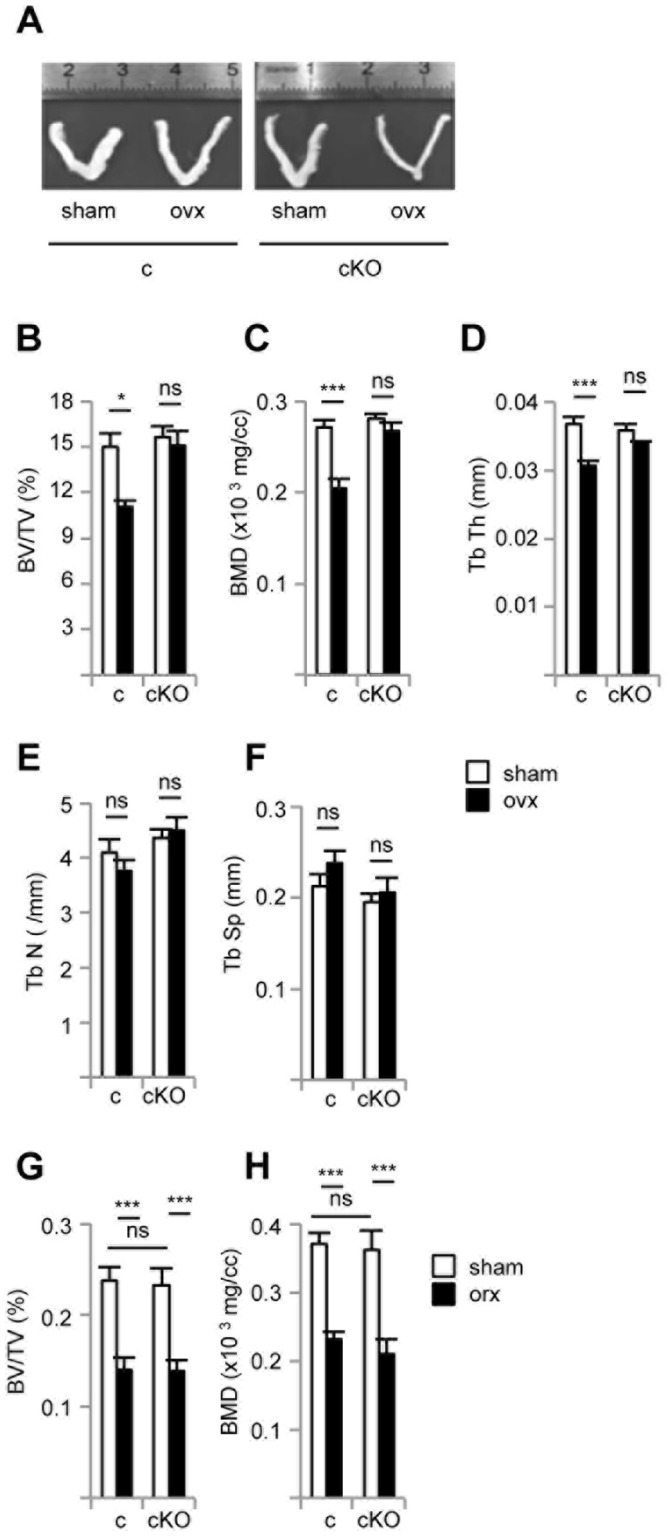
Conditional inactivation of ERRα *in vivo* protects against ovariectomy-induced bone loss. **A.** Photograph of uteri in sham-operated or ovariectomized (ovx) control (c) or cKO mice. **B–F**. Same parameters as in Fig. 3 were determined in females either sham-operated (white bars) or two weeks after ovariectomy (ovx; black bars). BV/TV (**G**) and BMD (**H**) were determined in male mice either sham-operated (white bars) or four weeks after orchidectomy (orx; black bars). All mice (n = 6 to 10 per group) were 14 wk old at the end of the experiment. Error bars represent s.e.m. *: *p*<0.05; ***: *p*<0.005; ns: non significant.

We next studied the bone structural parameters two weeks after gonadectomy on control and cKO animals (Fig. 5). Female mice of both genotype responded identically to ovariectomy (ovx) in terms of reduced uterine thickness (Fig. 5A). In control female mice, we observed the expected decrease in bone volume (Fig. 5B), bone mineral density (Fig. 5C) and trabecular thickness (Fig. 5F) upon ovx. As also expected the number of trabeculae was unchanged (Fig. 5D) and a non-significant trend toward enhanced spacing between trabeculae was observed (Fig. 5E). Strikingly, none of these parameters were modified in ovariectomized cKO mice as compared to sham-operated animals. The divergent response to ovx of control and cKO mice can also be viewed on the reconstructed 3D structures of trabecular bones (see Movies S1, S2, S3, and S4). This situation is in full contrast with the one prevailing in male mice in which orchidectomy (orx) led to dramatically reduced bone volume (Fig. 5G) and mineral density (Fig. 5H) both in control and cKO mice. We concluded that inactivating ERRα during osteoblast maturation completely protects against bone loss induced by hormonal deficiency, selectively in females.

## Discussion

Amongst other, ageing is associated with bone loss, leading to osteoporosis (understood as bone fragility syndrome) and increased fracture risk. Due to the global ageing of the human population worldwide, osteoporosis is now a major health problem, being the most common metabolic disorder of old age in humans [1], [2]. During ageing bone formation by osteoblasts is impaired due a decreased number and activity of osteoblasts, and concomitantly bone resorption by osteoclasts is increased. Whereas both males and females are subjected to age-associated osteoporosis, the situation is further aggravated in females after menopause. The cessation of ovarian function, resulting in highly decreased circulating levels of estrogens, indeed leads to unimpaired osteoclast differentiation and activity which is not compensated for by an equivalent rise in osteoblast activity [32]. Although age-related- and estrogen-deficiency-related bone loss essentially originates in different cell compartments, it is expected that enhancing bone anabolism (*i.e.* promoting bone formation by osteoblasts) could be an efficient mean to counteract osteoporosis in general [6], [7].

In this line, we and others have shown that genetic inactivation of the orphan nuclear receptor ERRα in mice leads to resistance to bone loss induced by ovariectomy (used as a model for estrogen-deficiency, thus mimicking menopause) or ageing [23], [24]. Interestingly these phenotypes were not associated with a decrease in osteoclast activity suggesting that ERRα does not modulate bone resorption *in vivo* at least under the above-mentioned challenging conditions. In contrast, ERRαKO animals displayed a higher bone formation rate as compared to wild type ones, altogether strongly suggesting that ERRα contributes to bone loss exclusively through the effects it exerts in osteoblasts. It should however be mentioned that ERRα has been shown to be required for osteoclast differentiation and/or activities in response to bone loss induced by rosiglitazone, a thiazolidinedione prescribed for the treatment of insulin resistance and diabetes [33].

The actual role of ERRα in osteoblast differentiation *in vitro* (inducer or inhibitor of differentiation?) is controversial and has been thoroughly discussed in our recently published review [27]. However, the absence of ERRα has been associated to an increased capacity of mesenchymal cells (MCs) to differentiate in osteoblasts *ex vivo* [23], [24]. Conversely MCs were less prone to differentiate into adipocytes in the absence of the receptor [22] leading to reduced fat marrow in ERRαKO animals [23] suggesting that ERRα is an early switch factor influencing MCs differentiation towards the adipocyte pathway at the expense of the osteoblastic one. ERRα has been suspected of additional effects in later steps of osteoblast maturation which may be mediated by osteopontin (opn), a downstream positive target of the receptor [30]. Indeed opn has been shown to reduce bone mineralization and its absence in KO mice confers resistance to ovx-induced bone loss [34], as does inactivation of ERRα.

The conditional KO approach described in the present report allows us to discriminate between the early (MCs commitment) and late (osteoblast maturation) effects of ERRα. To inactivate a floxed ERRα allele, we indeed expressed the Cre recombinase under the control of a Col1a promoter fragment, driving expression during mid stages of osteoblast differentiation, *i.e.* well after MCs commitment to the osteoblastic lineage [28]. As a consequence we observed a decrease in ERRα expression only during mid to late stages of osteoblast maturation *ex vivo*, whereas the receptor is normally present in pre-osteoblasts. Consistently the expression of Runx2, an early differentiation marker and inducer [8], was normally regulated during cKO pre-osteoblast differentiation in contrast to what observed in complete ERRαKO mice, where a marked increase of Runx2 expression had been observed [23], [24]. However, the expression of mid- to late markers of osteoblast differentiation was deregulated in cKO-originating cells, in a manner similar to that observed in complete ERRαKO animals. In this respect our data suggest that ERRα may reduce osteoblast differentiation by antagonizing Runx2 transactivation capacities, in a manner similar to that demonstrated for the closely related ERRγ receptor [26].

**Figure 6:**
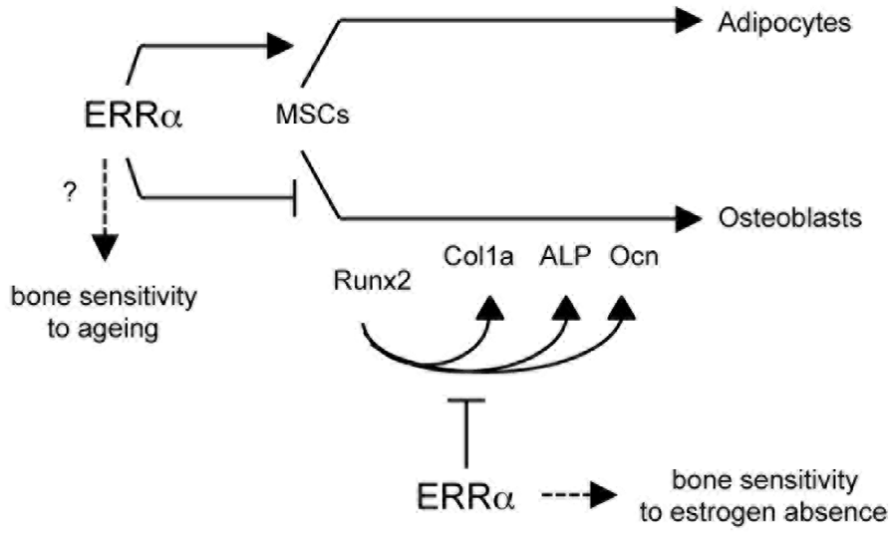
*In vivo* and *ex vivo* effects of ERRα on bone. ERRα impairs the later steps of osteoblastic differentiation. The results presented here show that these activities are involved in bone loss induced by estrogen deficiency and may involve the repression of Runx2- driven transcriptional activities. In contrast, these activities are not involved in bone sensitivity to ageing. Data published by other show that ERRα also promotes the early commitment of mesenchymal stem cells (MSCs) toward the adipocytic pathway while restricting the osteoblastic one. We hypothesize that these early activities are responsible, at least in part, for bone loss induced by ageing. See text for details and references.

Age-induced bone loss in cKO mice was similar to the one measured in control animals. This excludes that the activities of ERRα during osteoblast maturation may mediate age-dependent bone loss. It is tempting to rather speculate that the activities of ERRα as a repressor of MCs commitment to the osteoblastic lineage are involved in this phenomenon. However, we cannot formally exclude the impact of a yet uncharacterized primary activity of ERRα outside the bone compartment that would result in age-related bone loss. Investigating the bone response of ERRα cKO animals to gonadectomy revealed that males were normally sensitive whereas females were completely protected from bone loss. This situation is actually identical to the one observed in complete ERRαKO mice and confirms a gender-dependent effect of the receptor in bone. More importantly our results demonstrate that ERRα contributes to ovariectomy-induced bone loss via its activities on osteoblast maturation, and not through the regulation of MCs commitment. These results are summarized on Figure 6.

Together with data published by other laboratories, our results suggest that ERRα could be a promising target for the design of innovative therapies against bone loss, specifically in females. In this respect, we previously demonstrated that expression of the receptor in bone is not modified according to the estrogen status in mice [35]. Although ERRα is an orphan receptor, several synthetic compounds have been identified that modulate its activities and impact on its stability [10]–[14]. A pharmacological approach could thus be considered to specifically impact on the receptor. However complete inhibition of the receptor can be expected to lead to various undesired side effects on metabolism. For instance, given the role of ERRα as a switch factor in MCs commitment, its inhibition, while promoting osteoblast differentiation, would likely affect adipogenesis and thereby lipid storage and consumption. A more reliable approach would consist in impacting only the late osteoblast activities of the receptor, although this would solely be efficient against estrogen deficiency-induced bone loss. Such a compound, modulating a specific subset of the receptor’s activities, is still to be identified.

## Materials and Methods

### Animals

Col1a-Cre mice have been described elsewhere [28]. ERRα^fl/fl^ (Esrra<tm1ICS> mouse line) animals have been generated in the Institut Clinique de la Souris (Illkirch-Graffenstaden). For genotyping, DNA extracted from organs using conventional methods was PCR amplified using Eurobio kit. PCR cycle used: 94°C, 30 sec; 62°C, 30 sec; 72°C, 1 min. PCR products were analysed on 1.2% agarose gels.

### Primers Used for Genotyping

a: 5′-GCCCCCCTTGGCCCCCCTTAGCCCCCTCCC-3′
b: 5′-CCCTGCTTCTGTGCCCTTTGC-3′
c: 5′-CCACCACTGCCCAGCTTCAC-3′

For surgery, animals were anesthetized with sodium pentobarbital. Testes were ligatured and cut through an incision in the scrotum. Ovaries were removed through an incision in the flanks. Animals were sacrificed 2 wk after operation.

All animal experiments were performed in the Plateau de Biologie Expérimentale de la Souris (PBES; ENS Lyon) under animal care procedures, conducted in accordance with the guidelines set by the European Community Council Directives (86/609/EEC) and approved by the ENS Lyon ethical committee. Animals were in C57black6 background and had access to food and water *ad libitum*. Mice were sacrificed by cervical dislocation at 10 a.m.

### X-ray Microcomputed Tomography Analysis

3D microarchitecture of the femur was evaluated using a high-resolution (8 µm) microtomographic imaging system (eXplore Locus, GE, USA). A 3D region within the secondary spongiosa in the proximal metaphysis of the femur was reconstructed, beginning 500 µm proximal to the growth plate and extending to 1.5 mm. Cortical bone was reconstructed from a 1 mm thick region of interest centered on the diaphysis, 5 mm distal from the proximal growth plate. Morphometric parameters were computed using the Advanced Bone Analysis Microview Software (GE).

### Calvarial Cell Cultures

Calvariae were isolated from 1–5 day old mice and digested for 1 hr in collagenase (Sigma). After centrifugation, cell pellet was set in culture in six-well plates in αMEM supplemented with 10% fetal calf serum (FCS), 1% penicillin/streptomycin. Cells were induced to differentiate into osteoblasts with 50 µg/ml L-ascorbic acid and 10 mM β-glycerophosphate. Culture medium was replaced every 2 to 3 days. For immunofluorescence, cells were fixed in 4% paraformaldehyde for 10 min, then permeabilized with 0.5% Triton, 0.3% BSA in PBS for 5 min. Saturation was performed in 0.3% BSA in PBS for 30 min. Primary antibodies (anti-ERRα from Santa Cruz diluted 1/50; anti-collagen1 from Novotech diluted 1/200) were added or 3 hrs at RT. Secondary antibodies from Jackson (anti-mouse-Alexa488 diluted 1/1000) or Interchim (anti-rabbit-Alexa647 diluted 1/1000) were incubated for 1 hr at RT. Hoescht staining was diluted 1/20,000 and incubated for 10 min. For ALP staining, cells were rinsed with PBS, then fixed with 4% (v/v) formaldehyde for 5 min. After 3 washes with H2O, cells were stained using the alkaline phosphatase fast red violet kit (Sigma) following the indications provided by the manufacturer. Detection of mineralization activity was performed 15 days after the onset of differentiation. To this end cells were washed with PBS, then stained with 5% silver nitrate under UV irradiation for 10 min, then washed and dried.

For lentivirus construction, flagged human-ERRα fragment was inserted into the blunted BamHI site of the pRRL.PPT.SF.i2GFPp plasmid. This plasmid was provided by the Lentivus Production platform (ENS Lyon), which produced the recombinant lentivirus. Virus titers were determined upon infection of 293T cells. Calvarial cells were infected (MOI 5 in PBS) one day after induction of differentiation. Empty virus was used as a control.

### Expression Analysis

RNAs were purified using Guanidinium thiocyanate/phenol/chloroforme extraction. Total RNAs were reverse transcribed using iScript retrotranscription kit (Biorad). Quantitative PCR were performed using the iQ SYBR-Green kit (Biorad) in duplicate on a Biorad Cfx1000 apparatus using standard procedures. Results were analysed using the ΔΔCt method normalized to the expression of the 36b4 housekeeping gene.

### Sequence of the Primers Used for Real time PCR

36b4∶5′-ACCTCCTTCTTCCAGGCTTT-3′ and 5′-CCCACCTTGTCTCCAGTCTTT-3′; ALP: 5′-GCCCTCCAGATCCTGACCAA-3′ and 5′- GCAGAGCCTGCTGGTCCTTA-3′;

Col1a: 5′-CTGACGCATGGCCAAGAAGA-3′ and 5′-GCATACCTCGGGTTTCCACG-3′; ERRα: 5′-CAAACGCCTCTGCCTGGTCT-3′ and 5′-ACTCGATGCTCCCCTGGATG-3′; Ocn: 5′-ACCTCACAGATGCCAAGCCC-3′ and 5′-AGCGCCGGAGTCTGTTCACT-3′; Opn: 5′-TCTCCTTGCGCCACAGAATG-3′ and 5′-TCGTCCATGTGGTCATGGCT-3′; Runx-2∶5′-GACGTGCCCAGGCGTATTTC-3′ and 5′-GGAACTGCCTGGGGTCTGAA-3′.

For Western blot analysis, cells were lysed in RIPA buffer. 30 µg proteins were resolved on a 10% SDS-PAGE, blotted onto nitrocellulose membrane (GE-Healthcare) and probed with antibodies (ERRα from Genetex, hsp90 from Santa Cruz) after saturation.

### Analysis of Transcriptional Activities

C3H10T1/2 cells were cultured in MEM supplemented with 10% FCS. For transient transfection, 15.10^3^ cells were seeded in 96-well plates and transfection using 0.75 ml of ExGen500 (Euromedex), 12.5 ng firefly luciferase reporter plasmid (ERRE-Luc or OSE6-Luc) and the indicated amount of activator-encoding plasmids. 12.5 ng CMV-renilla Luciferase were added to normalize transfection efficiency and pSG5 plasmid was added as a carrier up to 125 ng. Cells were lyzed 48 h after transfection and reporter activities were determined using standard methods. All transfections were performed in triplicate. pSG-ERRα, pSG-ERRγ and ERRE-luc have been described in [36], CMV-Runx2 and 6OSE-Luc (generous gifts of Patricia Ducy) in [31].

### Statistical Analysis

Statistical significance was analyzed using one-way ANOVA.

## Supporting Information

Movie S1 **3D reconstitution of trabecular bone originating from control sham female mice.**
(AVI)

Movie S2 **3D reconstitution of trabecular bone using control ovx mice.**
(AVI)

Movie S3 **3D reconstitution of trabecular bone using cKO sham mice.**
(AVI)

Movie S4 **3D reconstitution of trabecular bone using cKO ovx mice.**
(AVI)
